# Sentinel Node Biopsy Alone versus Completion Axillary Node Dissection in Node Positive Breast Cancer: Systematic Review and Meta-Analysis

**DOI:** 10.1155/2014/513780

**Published:** 2014-10-14

**Authors:** Rachna Ram, Jasprit Singh, Eddie McCaig

**Affiliations:** ^1^Plastic Burns and Maxillofacial Unit, Hutt Valley DHB, Private Bag 31907, Lower Hutt 5010, New Zealand; ^2^Fiji National University College of Medicine, Nursing and Health Sciences, Private Mail Bag, Brown Street, Suva, Fiji; ^3^Colonial War Memorial Hospital, Private Mail Bag, Brown Street, Suva, Fiji

## Abstract

*Introduction*. There has been recent interest in validity of completion axillary node dissection after a positive sentinel node. This systematic review aims to ascertain if sentinel lymph node dissection alone was noninferior to axillary lymph node dissection for breast cancer patients who have a positive sentinel node. *Method*. A systematic review of the electronic databases Embase, MEDLINE, and Cochrane Register of Controlled Trials was carried out. Only randomised trials that had patients with positive sentinel node as the study sample were included in the meta-analysis using the reported hazard ratios with a fixed effect model. *Results*. Three randomised controlled trials and five retrospective studies were identified. The pooled effect for overall survival was HR 0.94, 95% CI [0.79, 1.19], and for disease free survival was HR 0.83, 95% CI [0.60, 1.14]. The reported rates for locoregional recurrence were similar in both groups. The surgical morbidity was found to be significantly more in patients who had underwent axillary dissection. *Conclusion*. Amongst patients with micrometastasis in the sentinel node, no further axillary dissection is necessary. For patients with macrometastasis in the sentinel node, it is reasonable to consider omitting axillary dissection to avoid the morbidity of the procedure.

## 1. Introduction

While sentinel node biopsy is becoming the standard of care in developed countries and survival rates improve, emerging data from studies have questioned the use of axillary lymph node dissection (ALND) in breast cancer patients with positive sentinel lymph node (SLN).

The general reasoning behind axillary dissection in breast cancer is that it is needed for local and regional control of the axilla as well as providing important information for adjuvant systemic therapy and prognostication.

The radical mastectomy by Halsted [1] and modified mastectomy by Patey and Dyson [2] were surgically designed to allow access to the axilla.

In 1971 NSABP B-04 [3] study challenged the Halsted theory by randomising 1665 women to different treatment arms. A 25-year follow-up of this trial continues to demonstrate no significant differences in long term outcomes between clinically negative-node patients who received radical mastectomy and those who received total mastectomy with or without irradiation [4].

Another landmark study, the NSABP B-06 trial [5] in 1976 randomised women with Stages I and II breast tumours ≤4 cm in size to lumpectomy and axillary node dissection with or without radiation versus modified radical mastectomy. The trial concluded that breast conservation surgery with breast irradiation in all patients and adjuvant chemotherapy in women with positive nodes was an appropriate mode of therapy provided the resection margins were clear.

The Halsted theory that cancer spread in a contiguous manner was termed anatomical and mechanistic in a 1980 David A. Karnofsky Memorial lecture by Dr Fisher [6]. Dr Bernard Fisher proposed the Fisher Alternative theory that breast cancer was a systemic disease.

While the morbidity and complications of axillary node dissection were evident from the time Halstead advocated the radical mastectomy, interest in potential avoidance of this procedure gained limelight with the discovery of the sentinel lymph node (SLN).

One of the first descriptions of sentinel lymph node biopsy was described by Gould et al. in 1951 during a total parotidectomy when a normal appearing node was noted at the junction of the anterior and posterior facial veins, which was reported as a lymph node with metastatic tumour under frozen section [7].

The concept of SLN is based on the principle that there is a predictable orderly pattern of lymphatic drainage to a regional lymph node basin and that there is a first lymph node that may function as filter for tumor cells [8].

Two types of breast cancer patients typically present to the physician with reference to the axilla; those who have a clinically palpable node in the axilla and those who have a clinically negative axilla.

If, on clinical, radiological, and possible cytological examination, there is no axillary involvement, the axilla is defined as clinically negative and hence is eligible to undergo SLN biopsy.

For those with clinically apparent axillary disease, an axillary dissection continues to be carried out.

According to the seventh edition of the American Joint Committee on Cancer (AJCC) manual, micrometastasis is defined as tumours greater or larger than 0.2 mm but no greater than 2 mm.

Macrometastasis includes nodes with more than 2 mm tumour deposits.

The NSABP B-32 trial [9] concluded that when the sentinel node is negative, no further axillary dissection was needed in clinically node negative patients.

Since ALND was previously advocated for staging and decision making purpose, positive SLN with micrometastasis or isolated tumour cells have also been the subject of research to see how it affects decision making on the use of adjuvant systemic treatment [10].

The AMAROS study (After mapping of the axilla: radiotherapy or surgery) [11] found no significant difference between the number of patients who had adjuvant chemotherapy or hormonal therapy suggesting that knowing or not knowing the extent of nodal involvement did not significantly affect the prescription of adjuvant systemic therapy.

The Z0011 [12] trial by The American College of Surgeons Oncology Group is a phase 3 multicentre trial that created controversy by challenging the use of ALND when the sentinel lymph node is positive.

The aim of this review is to ascertain if carrying out sentinel lymph node dissection alone was noninferior to proceeding with an axillary lymph node dissection in clinically negative breast cancer patients who had a positive sentinel lymph node.

## 2. Methodology

A systematic review and meta-analysis was carried out according to the Preferred Reporting Items for Systematic Reviews and Meta-analysis (PRISMA) guidelines [13].

A review protocol had been registered on PROSPERO (International prospective register of systematic reviews) with registration number CRD42013004464.

*Population*. Breast cancer patients with positive sentinel node.

*Intervention*. Sentinel node biopsy without completion axillary node dissetion.

*Control.* Completion axillary node dissection.

*Outcomes*. Disease free survival and overall survival were primary outcomes. Secondary outcomes were local recurrence rates and surgical morbidity.

### 2.1. Electronic Search

The Electronic databases MEDLINE, Embase, and Cochrane Register of Controlled Trials were searched. The MESH terms Breast cancer, sentinel node, and axillary dissection were used. No limits were placed on age, language of publication, and publication status.

The bibliographies of the relevant published studies were also manually searched.

### 2.2. Inclusion and Exclusion Criteria

Studies that had population as positive SLN and compared SLN biopsy alone with ALND were included. Only randomised controlled trials were included in the primary analysis. A secondary analysis included observational studies. Studies that included negative sentinel node, axillary radiation, or assessed decision making for adjuvant therapy and prognostication were excluded from this study.

### 2.3. Study Selection

A second independent reviewer also performed the search. The randomised trials were assessed with a score assigned for each item identified according to the CONSORT checklist [14].

The studies were assessed for risk of bias according to the Cochrane Handbook for Systematic Reviews of Interventions (Table 1).

Funnel plots for meta-analysis for overall survival and disease free survival were symmetrical.

### 2.4. Data Extraction

An independent piloted form was used to collect data from the trials. The author of one of the trials was contacted for further relevant information [15] to retrieve more information but was unable to provide outcome using the requested measure.

### 2.5. Outcome Measures

The primary outcome measure for this study was overall survival and disease free survival, reported as adjusted and unadjusted hazard ratios with confidence intervals as well as overall percentage outcome measures.

Where outcome was reported using Log Rank analysis, estimates hazard ratios were derived [15].

An estimate for hazard ratio for disease free survival for the study by Sol$\overset{´}{\text{a}}$ et al. [15] was done using method as described by Tierney et al. [16].

Secondary outcome measures were local recurrence rates and rates of surgical morbidities.

### 2.6. Statistical Analysis

The statistical software Revman 5.1 was used for data analysis [23]. Outcomes for overall survival and disease free survival were included in the meta-analysis. Data Type was entered as generic inverse variance. Inverse variance was used as the statistical method with a fixed effects analysis model.

A random effects analysis model was also used for comparison and results were found to be similar for both outcomes. The effect measure used was hazard ratio with 95% confidence intervals. The unadjusted hazard ratios with their confidence intervals for above outcomes were used and Revman 5.1 was used to derive log (hazard ratio), standard error, *Z*-score, *P* value, and variance to generate forest plots.

Heterogeneity was assessed by the following methods:

- examination of forest plots to ascertain overlap of confidence intervals;
- chi-square test of heterogeneity and degrees of freedom: if the value of the chi-square statistic was larger than the degree of freedom, it was concluded than there was evidence of heterogeneity. (The Cochrane Collaboration open learning material);
- *P* value of the chi-square analysis: a *P* value of more than 0.10 was assessed as heterogeneity being insignificant and hence it was deemed as acceptable to combine the studies [24];
- examination of *I*-Square value.

## 3. Results

### 3.1. Study Selection

A search of MEDLINE, Embase, and Cochrane Register of Controlled Trials resulted in 2933 results with additional 12 studies from search of relevant bibliographies.

Limits were placed to include “Humans” and “trials” and the search was narrowed to 550.

**Figure 1:**
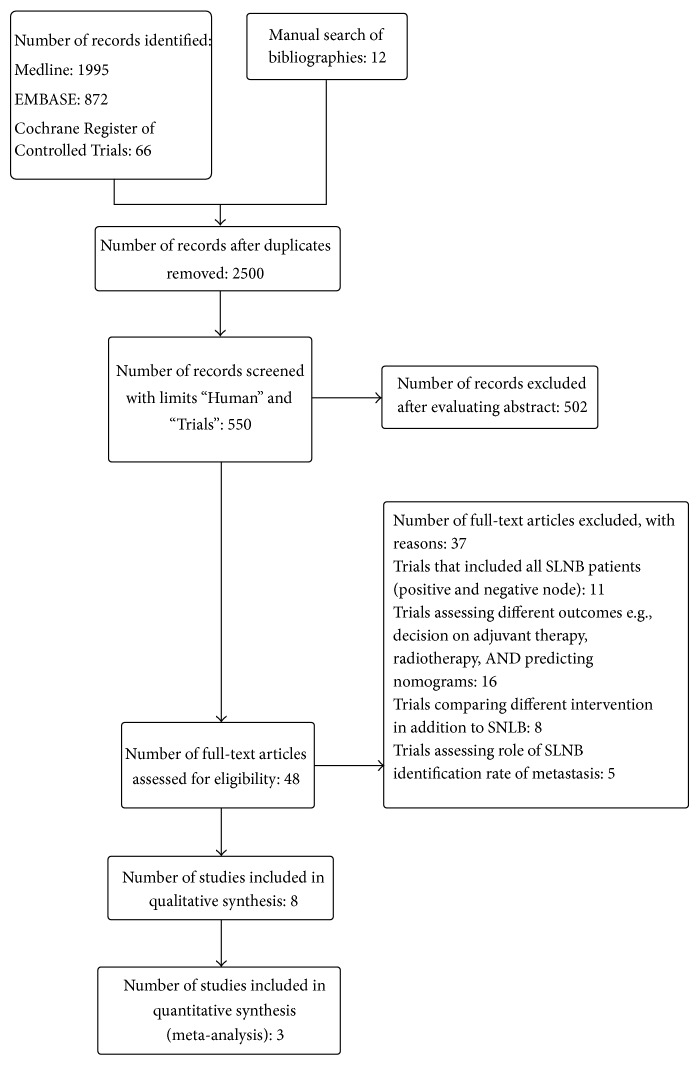
Flow diagram according to PRISMA statement [13].

After exclusion of 502 abstracts, 48 full articles were read (Figure 1). Three randomised trial (Table 2) and five retrospective studies (Table 3) were selected and included in this review.

### 3.2. Overall Survival Effect of SLNB Alone versus ALND

**Figure 2:**
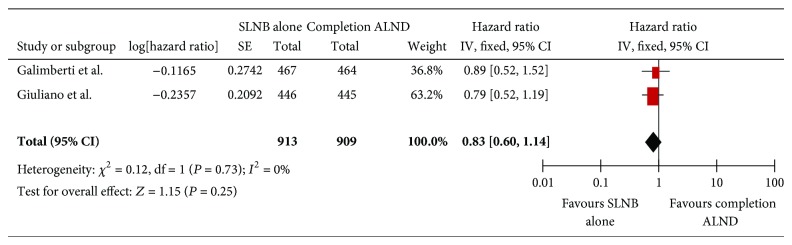
Forest plot showing pooled effect of overall survival in SLNB alone versus completion ALND in SLN positive breast cancer patients.

There was no significant benefit of sentinel lymph node biopsy alone over completion axillary node dissection (Figure 2).

Since one study did not have overall survival as the outcome of interest [15], only two trials were included in this analysis.

Visual inspection of the funnel plots suggested symmetry.

The chi-square test for heterogeneity suggested evidence of heterogeneity since the statistic was more than the degree of freedom. However, the *P* value of 0.73 suggested that this was not significant; hence a pooled overall effect was obtained. The overall *I*-squared statistic was 0.

The overall pooled effect suggested similar outcomes using both random and fixed effect models.

### 3.3. Disease Free Survival

Since the AATRM trial by Sol$\overset{´}{\text{a}}$ et al.[15] reported disease free survival outcome using the Log Rank test and Kaplan-Meier method, an estimate of hazard ratio was derived using method described by Tierney et al. [16].

**Figure 3:**
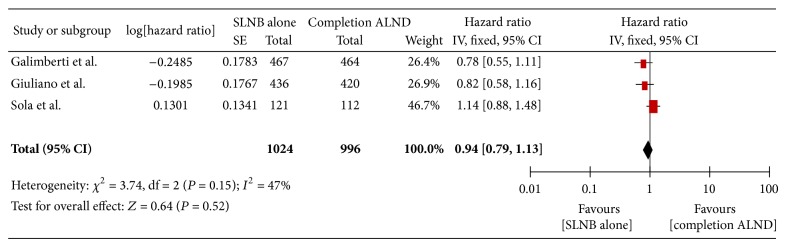
Forest plot showing pooled effect of disease free survival in SLNB alone versus completion ALND in SLN positive breast cancer patients.

There was no significant difference in SLN biopsy alone over ALND for patients with sentinel node metastasis (Figure 3).

Unadjusted hazard ratios were checked using both random and fixed effect model with no difference in outcome using either model.

Though the chi-square statistic in relation to degrees of freedom suggested evidence of heterogeneity, this was found to be not significant with a *P* value of 0.15 and thus the three studies were combined in a meta-analysis. The *I*-square statistic of 47% was also noted suggesting moderate heterogeneity.

### 3.4. Disease Recurrence

There were no significant differences in disease recurrence rates across the three studies (Table 3).

### 3.5. Surgical Morbidities

The rate of wound infections, axillary seromas, lymphoedema, motor neuropathy, and paresthesias was higher in the ALND group compared to SLN biopsy group (Table 3).

### 3.6. Secondary Analysis: Retrospective Studies of Breast Cancer Patients with Positive SLNB

Retrospective studies relevant to the study were identified (Table 4) including studies from the SEER (Surveillance, Epidemiology, and End Results) database [19] and the National Cancer Data Base [18].

The study from the SEER database by Yi et al. [19] looked at macrometastatic SLN and micrometastatic SLN separately. Lower locoregional recurrence rates were reported for patients with macroscopic SLN metastasis who underwent completion ALND. Although there was no statistical difference in survival data, the study concluded that omitting ALND in patients with macroscopic disease may result in higher regional recurrence.

The NCDB database study by Bilimoria et al. [18] noted that after analysis was adjusted for clinic-pathologic differences between the two groups, there was a trend for lower risk of recurrence and death for patients with macroscopic SLN who underwent ALND. However this was not statistically significant (Table 4).

## 4. Discussion

The overall findings from the randomised trials have shown noninferiority of SLND compared to ALND.

A recent systematic review and meta-analysis was identified during the search by Glechner et al. [25] which reported outcomes using odds ratios. A limitation of this study was that it included the Z0011 trial [12] with the two retrospective SEER study [19] and NCDB study [18] in a meta-analysis, resulting in large *I*-squared statistics and relatively long confidence intervals with significant heterogeneity.

A review by Francissen et al. [26] concluded that axillary recurrence rates were low in patients with a positive SLN without completion ALND and that omitting completion ALND was safe in patients with isolated tumour cells or micrometastasis.

At present this meta-analysis may be the first one to combine the Z0011 [12] trial with multicenter IBCSG 01 [17] and AATRM [15] trial with a combined pool data of 2020 patients.

The IBCSG 23-01 [17] and AATRM [15] trials are a welcome and timely addition to the gaps that were remaining after the Z0011 trial first created controversy and renewed interest in the topic of axillary dissection in SNL positive patients.

The NSABP-04 [3] trial which was started in 1976 had distant disease free and overall forty-six to forty-seven percent for different treatment arms. None of the patients had received adjuvant systemic therapy.

In this review, the randomised trials as well as retrospective studies have reported survival rates ranging from eighty-two to ninety-seven percent. All patients had systemic therapy compared to none in the NSABP-04 trial. This may be seen to reinforce the Fisher Alternative theory [27] that breast cancer is a systematic disease rather than the Halstedian concept of anatomical spread.

The Z0011 trial included only those breast cancer patients undergoing breast conservation surgery and all patients receiving whole breast irradiation whereas the IBCSG 23-01 trial included both mastectomy and breast conservative surgery patients.

The limitations of this study were that all three trials could not complete the planned accrual sample size due to the smaller than expected number of deaths and disease events. Factors that contribute to low accrual numbers include unwillingness of surgeons to consider foregoing ALND [28] as well as the patient's decision to be part of the trials. The higher than expected survival rates also affected the trials: even if the trial attained the planned accrual numbers, it could take up to 20 years of follow-up to observe the number of deaths needed to prove noninferiority [29]. There are not enough prospective studies or trials to address the specific issue of completion ALND in clinically node negative sentinel node positive patients as this is a difficult trial to achieve considering the limiting factors discussed. Only three randomised trials have been completed so far and all trials face similar limitations. A reason why inferiority was not found could be because two out of the three randomised studies [15] [17] had more micrometastatic sentinel nodes than macrometastatic: the micrometastasis could have been controlled by the adjuvant therapy as well as immune mechanisms.

There are concerns that the Z0011 results may have been confounded by whole breast radiation therapy causing incidental irradiation of the axilla [30]. Adjuvant therapy is not explained in the trial and could also have compensated for an undertreated axilla [30].

The Z0011 study included both micrometastatic and macrometastatic SNL while the IBCSG 23-01 trial only included patients with micrometastasis in SLN. All patients in IBCSG 23-01 received adjuvant systemic therapy.

The NCDB study by Bilimoria et al. [18] and SEER study by Yi et al. [19] analysed macroscopic disease and microscopic disease separately in positive SLN biopsy alone with a comparison cohort of patients who had completion ALND.

Overall survival rates reported for macroscopic sentinel metastasis were 82.1 versus 81.8%, *P* = 0.55 in the NCDB study [18], whereas the SEER [19] study showed that there were no differences in overall survival in macroscopic as well as in microscopic sentinel node metastasis in both treatment arms. The two studies noted a nonsignificant trend towards lower recurrence rates and death from patients with macroscopic sentinel node metastasis.

Studies by Galimberti et al. [20] and Guenther et al. [21] have also reported high survival rates.

The AATRM study [15], a multicenter funded trial carried out in Spain at 18 institutions, accrued patients with SNL micrometastasis only. However the study acknowledged that, prior to the updated definition of micrometastasis on SLN in the 6th Edition of the AJCC cancer staging manual, it had also included SLN isolated tumour cell. The exact number of patients with this was not specified.

There was no statistical difference in disease free survival. The unequal representation of micrometastasis, micrometastasis, and isolated tumour cells could account for the reason for moderate heterogeneity for disease free survival analysis.

The reported surgical morbidity of the axillary procedures was significantly worse in the ALND group compared to SLN biopsy alone (Table 3).

While two retrospective studies from the SEER and NCDB database [18, 19] had also reported a nonsignificant trend towards reduced axillary recurrence rates in ALND group of patients who had macrometastatic disease, this could not be ascertained in this meta-analysis.

## 5. Conclusion

For patients with a clinically negative axilla and micrometastasis in the SLN, this review shows that SLND alone is noninferior to completion ALND. The pooled effect for overall survival was HR 0.94, 95% CI (0.79, 1.19) and for disease free survival it was HR 0.83, 95% CI (0.60, 1.14). The reported rates for locoregional recurrence were similar in the SLND alone group compared to ALND. The surgical morbidity was found to be more in the ALND group compared to SLND alone.

Choosing SLN biopsy alone could avoid complications associated with ALND as the outcomes in terms of overall survival, disease free survival, and locoregional recurrence are similar in both.

For patients with macrometastasis to the axilla, we conclude that omitting ALND may also be considered a feasible option provided that the patients receive appropriate systemic chemotherapy and hormonal therapy. However, this should be considered with caution since this meta-analysis has a lower number of patients with macrometastatic sentinel node.

For developing countries that may not have access to the appropriate systemic therapy options including HER2/neu gene testing, ALND can still be considered.

## Figures and Tables

**Table 1:** Study quality.

Author	Random Sequence generation	Allocation concealment	Blinding	Blinding of outcome assessment	Incomplete outcome data	Selective reporting	Consort score
Giuliano et al. [12] Z0011	Yes	No	No	No	No	Yes	22
Galimberti et al. [17] IBCSG 23-01	Yes	Yes	No	No	Yes	Yes	22
Solá et al. [15] AATRM	Unclear	Unclear	No	No	No	Yes	18

**Table 2:** A characteristic of included randomised trials.

Study	Accrual period	Single/multicenter	Population	Sample size calculation	Follow-up	N SLNB alone/ALND	Primary outcome	Secondary outcome	Randomisation method	Adjuvant treatment
ACSOG Z0011 [12] trial	1999 to 2004	Multicenter in USA; 115 institutions	Adult women with histologically; confirmed invasive breast cancer 5 cm or less AND; breast conservation surgery AND; clinically negative axilla AND; positive metastatic SLN on frozen section, touch preparation, and H-E Stain (micrometastasis in SLND alone-164 ALND-137)	500 deaths needed for 90% power, 1900 patients accrual was planned, only 891 randomised	5.2–7.7 years; median 6.3 years	446/ 445	Overall Survival; occurrence of surgical morbidities	Disease free survival	Not mentioned	All received whole breast irradiation. Systemic therapy not specified

AATRM [15] Trial	2001 to 2008	Multicenter in Spain; 18 institutions	Patients with newly diagnosed breast cancer less than 3.5 cm AND; mastectomy or breast conservation surgery AND; clinically negative axilla AND; SLN micrometastasis with metastatic cell deposit 0.2–2 mm (from 2002) and excluding isolated tumour cells from 2002	352 patients planned for accrual based on survival curves with Log Rank method (247 recruited)	2 to 8.9 years; median 5.1 years	121/ 112	Disease free Survival		Not mentioned	All received postoperative systemic therapy. Breast-conserving surgery received tangential breast radiation

IBCSG 23-01 [17] trial	2001 to 2012	Multicenter from Europe, South America, and Australia; 27 institutions	Women newly diagnosed with breast cancer 5 cm or less AND; mastectomy or conservative surgery AND; clinically negative axilla AND; SLN micrometastasis including isolated tumour cells	558 events needed for 90% power, 1960 patients planned for target accrual (934 recruited)	3.6 to 7.3 years; median 5 years	469/ 465	Disease free Survival	Overall survival; site of recurrence; morbidity of ALND	Permuted blocks generated by a congruence algorithm	Majority of patients in both arms received systemic therapy and radiation therapy with breast conservation

**Table 3:** Summary of differences in outcomes of SLND alone versus ALND.

Study	Overall survival	Disease free survival	Local recurrence rate	Surgical morbidities
Giuliano et al. [12]	No difference; 92.5% versus 91.8%; *P* = 0.008	No difference; 83.9% versus 82.2; % *P* = 0.14	No difference; 1.6% versus 3.1%; *P* = 0.11	Benefit; 25% versus 70%; *P* < 0.001; worse in ALND group

Solá et al. [15]	Not assessed	No difference; 98.2% Log rank test; *P* = 0.330	No difference; 1.7% versus 1%; *P* = 0.348	Not assessed

Galimberti et al. [17]	No difference; 97.5% versus 97.6%; Log Rank *P* = 0.73	No difference; 87.8% versus 84.4%; Log Rank *P* = 0.16	No difference; low in SLNB <1%	Benefit; Sensory neuropathy, 12% versus 18%, *P*—0.012; motor neuropathy, 3% versus 8%, *P* = 0.0004; Lymphoedema, 3% versus 13%; *P* < 0.0001; worse in ALND group

**Table 4:** Summary of Findings of Included Retrospective Studies.

Author	Year	Population	Intervention SLNB alone	Control completion ALND	Follow-Up	Overall survival	Disease free survival	Locoregional recurrence
Bilimoria et al. [18] NCDB	1998–2005	SLNB positive *n*—97,314; macroscopic *n*—87,055; microscopic *n*—10,259	20,217 Macroscopic *n*—16,543 Microscopic-3,674	77,097; macroscopic *n*—70,512; microscopic *n*—6,585	63 months	Macroscopic, HR, 0.89, 95% CI, 0.76–1.04, 82.1 versus 81.8%,, *P* = 0.55; microscopic, 90.3 versus 90.3%, *P* = 0.98; no significant difference	Macroscopic, 89.9 versus 89.1%, *P* = 0.18, microscopic, 99 Versus 97.8%, *P* = 0.81; no Significant difference	Macroscopic, 1.0 versus 1.2%, *P* = 0.40; microscopic, 0.4 versus 0.2%, *P* = 0.18; lower in ALND but not significant

Yi et al. [19] SEER database	1998–2004	SLN Positive *n*—26,986; macroscopic *n*—20,146; microscopic 6,838	4,425 Macroscopic *n*—2,185 Microscopic *n*—2,240	22,561; macroscopic *n*—17,963; microscopic *n*—4,598	50 months	Macroscopic, HR 1.2, 95% CI, 1.1–1.4; overall no significant difference including microscopic SLN	Macroscopic, HR 1.5, 95% CI, 1.3–1.8; overall no significant difference including microscopic SLN	Microscopic, 0.2 V 0.08%, HR—0.30, *P* = 0.02; macroscopic: lower risk in ALND but not significant; microscopic: no difference

Galimberti et al. [20]	1999–2007	SLN microscopic *n*—377	377	None	60 months	97.3% (95% CI, 95.3–99.3)	Not mentioned	2.4% (95% CI, 1.1–4.5)

Guenther et al. [21]	1996–2001	SLN positive *n*—46	46	None	32 months	100%	97.8%	None

Spiguel et al. [22]	1998–2009	SLN positive *n*—123	123	None	94.8 months	Not mentioned	85%	0.8% axillary, 1.7% breast recurrence
